# A comparision of the yield of three tuberculosis screening modalities among people living with HIV: a retrospective quasi-experiemental study

**DOI:** 10.1186/s12889-016-3763-9

**Published:** 2016-10-13

**Authors:** Michael Kakinda, Joseph K. B. Matovu, Ekwaro A. Obuku

**Affiliations:** 1 Ministry of Health, Plot 6 Lumumba Avenue, P.O. Box 7061, Kampala, Uganda; 2 Makerere University College of Health Sciences, School of Public Health, P.O. Box 7072, Kampala, Uganda; 3 Clinical, Operational and Health Services Research, Joint Clinical Research Centre, P.O. Box 10005, Kampala, Uganda; 4 Makerere University College of Health Sciences, School of Medicine, P.O. Box 7072, Kampala, Uganda; 5 Faculty of Epidemiology and Population Health, London School of Hygiene and Tropical Medicine, WC1E 7HT London, UK

**Keywords:** Intensified case finding, Uganda, HIV/AIDS

## Abstract

**Background:**

The Intensified Case Finding (ICF) tool was approved for TB screening in 2011; however there is still paucity of robust data comparing yields of the different ICF screening modalities. We compared yields of three different screening modalities for TB among Patients Living with HIV (PLHIV) in Uganda in order to inform National TB Programs on the most effective TB screening method.

**Methods:**

This was a retrospective quasi-experimental study conducted at an Out-Patient HIV/AIDS clinic in Uganda. We set out to determine yields of three different TB screening modalities at three time periods: 2006/07 where Passive Case Finding (PCF) was used. Here, no screening questions were administered; the clinician depended on the patient’s self report. In 2008/09 embedded Intensified Case Finding Tool (e-ICF) was used; here a data capture field was added to the patient clinical encounter forms to compel clinicians to screen for TB symptoms. In 2010/11 Independent Intensified Case Finding Tool (i-ICF) was used; here a screening data collection form, was used, it had the same screening questions as e-ICF. Routine clinical data, including TB status, were collected and entered into an electronic clinical care database. Analysis was done in STATA and the main outcome estimated was the proportional yield of TB cases for each screening modality.

**Results:**

The overall yield of TB cases was 11.18 % over the entire period of the study (2006 – 2011). The intervention–specific yields were 1.86 % for PCF, 14.95 % for e-ICF and 12.47 % for i-ICF. Use of either e–ICF (OR: 9.2, 95 % CI: 4.81-17.73) or i– ICF (OR: 7.7, 95 % CI: 4.02-14.78) significantly detected more TB cases compared to PCF (P <0.001). While the yields of the Active Case Finding modalities (e-ICF & i-ICF) were not significantly different (OR: 0.98, 95 % CI 0.76-1.27, *P* = 0.89).

**Conclusion:**

The active screening modalities (e-ICF & i-ICF) had a comparable TB yield and were eight to nine times more efficient in identifying TB cases when compared to the PCF. Cost effectiveness studies would be informative.

## Background

The World Health Organization’s (WHO) directly observed short course strategy (DOTs) was intended to achieve a treatment success rate (TSR) of >85 % and a case detection rate (CDR) of >70 % with a view to decreasing Tuberculosis (TB) incidence by 11 % annually [1]. However, despite the improvement in TSR from 43 % in 1994 [2] to 86 % in 2013 [3], CDR has stabilized at around 60 % since 2005 [4] and TB incidence remains generally higher in most low and middle-income countries [5]. Several reasons have been advanced to explain this phenomenon including the HIV pandemic and use of passive case finding approach which rely on waiting for patients to self report the signs and symptoms of TB. Recent TB prevalence surveys have suggested that there is a high burden of undiagnosed TB [6–9]. This is corroborated by the fact that only 6 million new TB cases were reported to WHO in 2014, out of an estimated 9.6 million TB patients globally [3]. Subsequently TB incidence has only decreased by only 1 % annually and not the anticipated 11 % [4]. Thus, the DOTs strategy which mainly uses passive case finding approaches has failed TB control efforts especially in countries which have a generalized TB epidemic (prevalence greater than 1 % of the general population) [10, 11].

In recognition of this failure, WHO recommended use of the intensified case finding tool for TB screening in 2004 in order to improve TB case detection or TB yield especially among high-risk persons such as persons living with HIV [12]. The different ICF modalities have been used in isolation, and they have been associated with an increased yield of TB patients [13–17]. However there is paucity of robust data trying to compare different ICF modalities in the same setting.

We set out to compare the yield of three screening modalities among people living with HIV (PLHIV) in a peripheral health facility in Uganda. These screening modalities were: (i) Passive case finding (PCF), where no screening questions were administered and the clinicians depended on the patients’ signs and symptoms, (ii) embedded intensified case finding (e-ICF), where a data capture field was added to the patient encounter forms to compel clinicians to screen for TB, and (iii) independent intensified case finding (i-ICF) a data collection form was utilized. The form had the same screening questions as e-ICF except that it was a standalone A4 form. The primary aim of the study was to identify which screening modality had the best yield.

## Methods

### Study design and setting

We conducted a retrospective quasi-experimental study of People Living with HIV (PLHIV) attending a Private Not for Profit (PNFP) HIV/AIDS clinic offering comprehensive services in Jinja district, Eastern Uganda. The HIV clinic was started in September 2004 following with the PEPFAR initiative.

### Study population

The study population was HIV-positive patients enrolled in HIV care between 2006 and 2011. Patients that were found to be HIV positive were enrolled into the clinic and then they were treatment according to the Ugandan HIV/AIDS treatment guidelines [18]. By 31st December 2011, the clinic had 2800 PLHIV clients registered in care and alive; 1,040 of these (37.14 %) were males, 313 (11.17 %) were children under the age of 14 years and 1,934 (69.07 %) were on Antiretroviral therapy (ART).

Over the study period the clinic had two doctors, four Nurses, two Laboratory, two Counselors, two data clerks, two pharmacy technicians and a receptionist, the same staff was kept over the whole study period. All the patients who came to the clinic got their files from the reception and were directed to the Nurse in-charge for the assessment of vital signs. If the patient had no complaints, they were sent to another nurse who gave them a drug refill. The patients were allowed to get a drug re-fill without seeing the doctor twice but it was a must every 6 months. Here the patients had their vitals taken by the nurse, who sent them to the doctor. The doctor assessed the patient and also requested for laboratory tests mainly a Cluster for Differentiation 4 (CD4), Liver Function Tests (LFT’s) and Renal Function Tests (RFT’s). Then after passing by the laboratory, the patient would get the medicine from the pharmacy. However, if the patient was very ill, the patient was sent to the doctor immediately. The doctor then did the vitals as well as assessing the patient and offered the necessary care and treatment. Patients were often given bi-monthly appointments. Any patient who missed their appointment was given a call the following day. A home visit was done if the patient did not return to the clinic two weeks after the appointment. This was done to ascertain why the patient missed their appointment. But otherwise patients were allowed to visit the clinic any day in case of emergencies. Sometimes the patient was sent to the counselor but was mainly for either patients who are starting Anti-retroviral Treatment (ART) or those who were thought to be non-adherent. TB screening could be done by any staff along the process.

### Intervention description

Three different strategies were used to identify potential TB patients: passive case finding, embedded intensified case finding, and an independent paper-based intensified case-finding tool, the different modalities were used exclusively. Whenever there was a change in intervention, the clinic staffs were taught about the new intervention at the same time, and they were oriented on the new tools to use.

With Passive Case Finding (PCF), no screening questions were administered since the identification of suspected TB cases was based on patients’ self-reports of any of the TB-related symptoms (i.e., current cough, fever, weight loss or night sweats) without prompting by a clinician. This was the standard of care between 2006 and 2007.

Between 2008 and 2009, a data capture field was added to the patient clinical encounter forms to compel clinicians to screen for TB symptoms for each patient. The data capture field necessitated the clinician to ask if the patients had any of the following symptoms and signs, current cough, drenching night sweats, night fevers, unexplained weight loss > 10 % of the body weight, and all respondents reporting any of the above-mentioned TB-related symptoms were considered for full TB evaluation. This underlies what we referred to as the embedded Intensified Case Finding (e- ICF) strategy.

Finally, the National TB and Leprosy Program of Uganda (NTLP) in collaboration with the AIDS Control Program (ACP) introduced a screening data collection form on which details and symptom questionnaire results are recorded each time a PLHIV attends the clinic (independent paper based Intensified Case Finding tool (i-ICF)). This i-ICF approach used the same screening questions as the e-ICF.

### Inclusion and exclusion criteria

We included all patients who had at least one clinic visit between 1st January 2006 and 31st December 2011 (6 years).

The following patients were excluded, patients known to have TB or on TB treatment and cases missing significant variables. These were Basal Mass Index (BMI) ≤ 15 or ≥ 40, Cluster for differentiation 4 (CD4^+^) > 1500 cells/mm^3^ and were the age variable could not be determined.

### Data collection methods and procedures

During patient visits clinic staff prospectively entered into an electronic database the clinical parameters, ART initiation and adherence, WHO stage, toxicities, and opportunistic infections.

We extracted data of all the patients who had a cough for two weeks or more, fevers, weight loss or night sweats. We then corroborated this data with the health facility paper–based TB recording and reporting tools of the Uganda NTLP. These were: the TB laboratory registers for persons undergoing sputum examination for suspected TB; the unit TB treatment register and TB pharmacy dispensing logs for those who received treatment for TB. Where data was unclear or inconsistent, we validated these by reviewing clinical notes from the patient charts.

### Outcome measures

The study main outcome of interest was yield of tuberculosis on using different screening modalities. The TB yield was defined as a percentage of the patients that were found with TB when a particular screening modality was used. The screening modality used was dependant on the time period. The different yields were determined by getting a proportion of patients with TB indentified by a particular TB screening modality over the number of active patients when the modality was used.

We defined a patient with Tuberculosis according to the guidelines of the Uganda NTLP as follows: a person with a sputum smear microscopy result positive for TB or a patient with signs and symptoms of Tuberculosis with a chest–radiograph suggestive of TB or a patient with signs and symptoms of Tuberculosis who is diagnosed to have Tuberculosis by a competent Medical Officer [19].

Patients that were identified as TB suspects by the various screening modalities were sent for further investigations. The investigations often used were sputum microscopy, chest radiography, abdominal ultrasonography, lymph node biopsy and Fine needle aspirate for microscopy. Mycobaterial culture and Xpert MTB/RIF tests were either not used because of the high costs or not being readily available at the time. The diagnosis was done based on the investigation and occasionally on clinical presentation alone.

### Data analysis

We exported the data from spreadsheet (Microsoft Excel version 2010) into Intercooled Stata version 11.2 (StataCorp, College Station, Texas, USA) for a complete cases analysis cases missing significant variables were excluded). We summarized demographic and outcome data into frequencies, percentages and measures of central tendency the median and mean. We explored the association between baseline characteristics and TB by univariate analysis (logistic regression) with crude odds ratios (OR) and 95 % confidence intervals. Statistical tests for association were two–sided and considered significant if *P* < 0.05. We included potential determinants of TB with *P* < 0.25 at univariate level, in a multivariable logistic regression model to estimate their adjusted odds ratios (aOR). In the time–to–event analysis were the start and end point was the beginning of a two year period when a screening modality was used, the censorships were death, lost to follow up, having TB or being on TB treatment. We compared the TB Case Detection Rate (CDR) of the screening interventions across the distinct cohorts enrolled during the three study periods: between 1st January and 31st December of 2006 to 2007, 2008 to 2009 and from 2010 to 2011.

The yield per screening modality was calculated using the number of TB cases detected over the number of active patients at the time. We then compared the yield of PCF to both e-ICF and i-ICF and that of e-ICF to i-ICF. The Case Detection Rate per 1,000 person years of observation (PYO) was also computed, and its corresponding 95 % confidence interval using the Wald statistic. We plotted survival curves using the Kaplan–Meier technique to depict this difference in yield of TB using the three different screening strategies. We report our findings in accordance with ‘The Reporting Quality of Non Randomized Evaluations of Behavioral and Public Health Interventions’ (TREND) statement [20].

## Results

### Socio-demographic and clinical characteristics of the patients

**Fig. 1:**
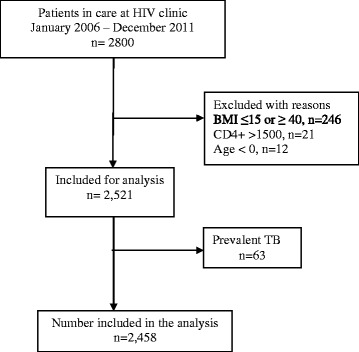
Flow diagram of study participants

Between 2006 and 2011, a total of 2800 patients were enrolled in HIV care at the HIV care clinic in Jinja district, Eastern Uganda. Of the 2800, 342 patients (63 patients had prevalent TB, while 279 had extreme variables, 246 had a BMI ≤ 15 or ≥ 40, while 21 had a CD4^+^ > 1500 cells/mm^3^ and 12 had an age <0 years of age) were excluded, leaving 2458 patients who were considered for the final analysis (Fig. 1). The cohort had 535 patients between 2006/07, 876 patients between 2008/09 and 1047 between 2010/11. The mean age was 34.8 years (SD: 11.5). Majority of the patients (61.2 %) were females and 149 (6.06 %) had a formal education (Table 1). The baseline median CD4+ cell count was 505 cells/mm3 (IQR 200 – 720 cells/mm3), while the mean BMI was 24.9 (SD: 5), and 1,912 (78 %) PLHIV were on ART. Participants diagnosed with Tuberculosis were more likely to be older (aOR: 1.02, 95 % CI: 1.01 – 1.04, *p* < 0.001), have a tertiary education (aOR: 2.38, 95 % CI: 1.41 – 4.04, *p* = 0.001), to be on ART (aOR: 3.38, 95 % CI: 2.16 – 5.32, *p* < 0.001) and have a higher BMI (aOR: 1.05, 95 % CI: 1.02 – 1.07, *p* < 0.001), (Table 1). Differences in the CD4+ cell counts were not statistically significant (*p* = 0.034).

**Table 1:** Factors associated with tuberculosis among PLHIV between 2006 and 2011

Variable	All cohort	TB	No TB	Univariate			Multivariate		
	*N* = 2,458 (100)	*n* = 275 (11.18)	*n* = 2,183 (88.81)	OR	95 % CI	*P*–value	aOR	95 % CI	*P*–value
Age (years)									
Mean (sd)	34.8 (11.5)	38.0 (11.7)	34.4 (11.5)	1.02	1.01 – 1.04	<0.001	1.03	1.01 – 1.03	<0.001
Gender									
Female	1,505 (61.2)	175 (63.6)	1,329 (60.9)	1		0.38			
Male	953 (38.7)	100 (36.4)	854 (39.1)	1.13	0.87 – 1.46		-	-	-
Education^#^									
None	501 (21.8)	40 (15)	461 (22.7)	1		<0.001	1		
Primary	993 (43.2)	134 (50.4)	859 (42.3)	1.80	124 – 2.61		1.72	1.18 – 2.51	0.005
Secondary	654 (28.5)	62 (23.3)	592 (29.1)	1.20	0.79 – 1.83		1.22	0.80 – 1.86	0.350
Tertiary	149 (6.5)	30 (11.3)	119 (5.9)	2.90	1.73 – 4.86		2.38	1.41 – 4.04	0.001
Missing	161 (6.5)	9 (3.27 %)	152 (6.96 %)						
ART Status									
No	546 (22)	22 (8)	524 (24)	1		<0.001	1		
Yes	1912 (78)	253 (92)	1659 (76)	3.64	2.32–5.71		3.38	2.16 – 5.32	<0.001
Baseline CD4+									
Median (IQR)	505 (200–720)	552 (286–782)	496 (192–712)	1.00	1.00.–1.00	<0.01	1.00	1.00 – 1.00	0.034
BMI									
Mean (sd)	24.9 (5)	26.1 (5.3)	24.2 (4.9)	1.05	1.03–1.08	<0.001	1.05	1.02 – 1.07	0.001

*Abbreviations*: *ART* antiretroviral therapy, *aOR* odds ratio adjusted for all variables with *p* < 0.25, *BMI* basal mass index, *CD4+* cluster of differentiation, *CI* confidence interval, *OR* crude odds ratio, *IQR* inter quartile range, *sd* standard deviation, *TB* Tuberculosis, *Significance level of *p* < 0.05, *()* refers to percentage unless stated

### Yield of Tuberculosis cases by screening modality

When PCF was introduced in 2006, the yield of TB was 1.87 % (10/535) increasing to 14.95 % (131/876) when e–ICF was introduced in 2008. The yield then decreased to 12.79 % (134/1,047) when i–ICF was introduced in 2011. Each of the modalities was used for a period of two years. This translated into a TB Case Detection Rate (CDR) of 15.4/1,000 PYO (95 % CI 5.9 – 24.9) for PCF, 137.8/1,000 PYO (95 % CI 115.8 – 159.7) for e-ICF and 123.9/1,000 (95 % CI 104.3 – 143.6) for i–ICF, respectively (Table 2).

**Table 2:** TB yield per screening modality

TB screening modality	*N* (%)	TB cases (%)	TB Yield	Time at risk^a^	CDR^b^	95 % CI
Passive Case Finding	535 (21.8)	10 (3.7)	1.86 %	647	15.4	5.9 – 24.9
Embedded – ICF	876 (35.6)	131 (47.6)	14.95 %	951	137.8	115.8 – 159.7
Independent – ICF	1047 (42.6)	134 (48.7)	12.47 %	1081	123.9	104.3 – 143.6
Any screening modality	2458 (100)	275 (100)	11.18 %	2679	102.7	91.2 – 114.1

*Abbreviations*: *ICF* intensified case finding, *CI* confidence interval, *TB* tuberculosis, *N* number of patients screened, *PYO* person years of observation

^a^Time at risk is in person years of observation

^b^Case detection Rate per 1000 person years of observation

**Fig. 2:**
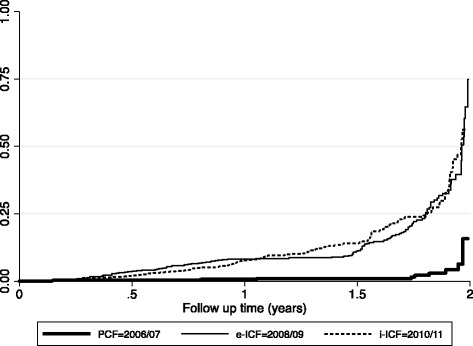
Graph showing the Cumulative Hazard curves of the three TB Screening modalities

This increment in the yield of TB was statistically significant when either e–ICF (OR: 9.2, 95 % CI: 4.81-17.73, *P* = 0.0001) or i–ICF (OR: 7.7, 95 % CI: 4.0-14.78, *P* = 0.0001) were introduced. However, there was no statistically significant difference in the yield of TB when i-ICF was compared to e-ICF (OR: 0.98, 95 % CI: 0.76-1.3, *P* = 0.89). These findings are further illustrated in Fig. 2.

## Discussion

The purpose of this study was to compare the yield of three different screening modalities among People Living with HIV (PLHIV). We found that either Active Case Finding modality (e-ICF or i-ICF) had a similar yield but it was 8-9 fold higher to the TB yield when PCF was used.

Use of any Active Case Finding (ACF) method for screening for TB, had a higher yield compared to the passive method (PCF). The better performance of active case finding strategies in our study in comparison to the Passive Case Finding corroborates results of other studies done in Uganda [14, 16, 17, 21, 22] which showed a higher yield of Tuberculosis with active screening. Our study is also consistent with a systematic review by Kranzer and colleagues [13] that found a median prevalence of 8.6 % (3.6 % - 24.7 %) in antiretroviral clinics in sub-Saharan Africa. We therefore call for the extensive implementation of the ICF tool for screening for TB, this will not only increase the Case Detection Rate but patients will be indentified early before infecting a lot more people.

Using of e-ICF for TB case finding was found with a comparable yield to using a standalone ICF form for screening. This is one of the few studies to compare 2 active screening modalities. The use of independent ICF form raises concerns of operational feasibility such as stock outs and patients being screened but records lost. We recommend the embedding of screening tools into the patient clinical encounter forms not only for TB but also other diseases, such as diabetes, were joint screening initiatives have proven effective [23–28].

Despite the TB yields of the two Active Case Finding modalities being comparable. That of e-ICF was 2.48 percentage points higher than i-ICF. We theorize that since in e-ICF the Intensified Case Finding Tool is embedded into the patient encounter forms. It was easier for the health workers to screen almost all patients they had an encounter with.

### Limitations

Our retrospective quasi-experimental study was not without limitations particularly the completeness and integrity of the data set which we circumvented by corroborating information from patient clinical notes and excluding missing data. We did not adjust for differences in time on HIV care, since longer duration on ART has been found to be protective. However, we adjusted for age, sex, CD4+ cell counts, body mass index and education status. Additionally, there could have been a detection bias due to the use of sputum smear microscopy, which has even lower sensitivity in patients with HIV, could have led to the underestimation of the burden of TB in this cohort particularly between 2006 and 2007 with very few events. Nonetheless, sputum smear microscopy remains the mainstay of TB diagnosis in peripheral HIV clinics due to implementation difficulties of scaling up the more robust Xpert MTB/RIF as per WHO recommendations [29].

## Conclusion

The active screening modalities (e-ICF & i-ICF) had a comparable TB yield but were eight to nine times more efficient in identifying TB cases when compared to the PCF. Therefore either case finding modality can be used for screening for TB. Embedding the ICF form into the patient’s clinical encounter form is more feasible. Future research could examine operational issues such as acceptability by health providers particularly in the private sector and logistical considerations of the ICF strategy. We also recommend same assessment but using diagnostic modalities with a better sensitivity and specificity like Xpert MTB/RIF.

## Abbreviations

ACF: Active case finding
ACP: Aids control program
ART: Antiretroviral therapy
BMI: Basal mass index
CDR: Case detection rate
CI: Confidence interval
e-ICF: Embedded-intensified case finding tool
i-ICF: Independent-intensified case finding tool
NTLP: National TB and leprosy program
OR: Odds ratio
P: Probability
PCF: Passive case finding
PLHIV: People living with HIV
PYO: Person years of observation
WHO: World Health Organization
